# An Adenosine Triphosphate- Dependent 5′-3′ DNA Helicase From sk1-Like *Lactococcus lactis* F13 Phage

**DOI:** 10.3389/fmicb.2022.840219

**Published:** 2022-03-15

**Authors:** Magdalena Chmielewska-Jeznach, Kamil Steczkiewicz, Kamil Kobyłecki, Jacek K. Bardowski, Agnieszka K. Szczepankowska

**Affiliations:** Institute of Biochemistry and Biophysics Polish Academy of Sciences, Warsaw, Poland

**Keywords:** *Lactococcus lactis*, sk1-like bacteriophage, P-loop NTPase, DNA unwinding, DNA replication

## Abstract

Here, we describe functional characterization of an early gene (*gp46*) product of a virulent *Lactococcus lactis* sk1*-*like phage, vB_Llc_bIBBF13 (abbr. F13). The GP46_*F*13_ protein carries a catalytically active RecA-like domain belonging to the P-loop NTPase superfamily. It also retains features characteristic for ATPases forming oligomers. In order to elucidate its detailed molecular function, we cloned and overexpressed the *gp46* gene in *Escherichia coli*. Purified GP46_*F*13_ protein binds to DNA and exhibits DNA unwinding activity on branched substrates in the presence of adenosine triphosphate (ATP). Size exclusion chromatography with multi-angle light scattering (SEC-MALS) experiments demonstrate that GP46_*F*13_ forms oligomers, and further pull-down assays show that GP46_*F*13_ interacts with host proteins involved in replication (i.e., DnaK, DnaJ, topoisomerase I, and single-strand binding protein). Taking together the localization of the gene and the obtained results, GP46_*F*13_ is the first protein encoded in the early-expressed gene region with helicase activity that has been identified among lytic *L. lactis* phages up to date.

## Introduction

Bacteriophages infecting the *Lactococcus lactis* species pose a great threat to industrial settings by perturbing or arresting milk fermentation processes (Garneau and Moineau, 2011). To elucidate the persistence of especially lytic lactococcal dairy phages, they have been quite extensively studied in terms of genome content and physio-morphological features (Deveau et al., 2006; Muhammed et al., 2017; Oliveira et al., 2018; Marcelli et al., 2020). Yet still, certain aspects of their biology, including genome replication, demand detailed characterization. The knowledge on the replication of lytic lactococcal phages and proteins participating in the process is of great value and may lead to developing new phage resistance mechanisms in lactococcal starter strains for controlling phage multiplication in dairy environments.

DNA replication is one of the pivotal processes in bacteriophage development. Distinct phages follow various replication pathways and exploit different sets of own and/or host proteins for multiplication of their genomic content (Weigel and Seitz, 2006 and references within). For example, the Gram(−) phage T4 and the Gram(+) phage phi29 possess all the necessary components of their replication machinery (i.e., initiator protein, primase, helicase loader, helicase, and DNA polymerase) (Meijer et al., 2001; Kulczyk and Richardson, 2016). In turn, other phages, such as *Escherichia coli* phage λ or *Bacillus subtilis* phage SPP1 encode their own replication initiation proteins, but depend on host proteins for further stages of the process. Specifically, proteins GP38, GP39, and GP40 of *B. subtilis* phage SPP1 execute the function of an origin initiation protein, helicase loader, and DNA helicase, respectively, and phage λ encodes only two proteins participating in replication–an origin-binding protein (protein O) and a helicase loader (protein P) (Mensa-Wilmot et al., 1989; Seco et al., 2013). Due to the generally restricted genome sizes, phage replication initiation functions quite often are coupled in one multi-activity protein, e.g., a primase-helicase like the GP4 protein of T7 and protein α of phage P4 (Bernstein and Richardson, 1988; Ziegelin et al., 1993), or the primase-polymerase of the deep-sea vent phage NrS-1 (Guo et al., 2019) and the corynephage BFK20 replication protein GP43 (Halgasova et al., 2012).

Studies on the replication of lactococcal phages and proteins engaged in the process have been limited essentially to temperate P335-group phages (McGrath et al., 1999; Ostergaard et al., 2001; Labrie et al., 2008). Phage TP901-1 ORF13 and phage Tuc2009 ORF16 (or Rep2009) were identified as the replisome organizer proteins that bind to regions rich in direct repeats located within their cognate genes and determined to serve as the origin region of phage replication (ori). Both elements, ori region and replisome organizer proteins, were shown to be directly engaged in phage replication and confer a PER (phage-encoded resistance) phenotype when cloned *in trans*. Sequence similarity searches performed for another P335-type phage, r1t, identified ORF11, and ORF12 as a homolog of the Rep2009 replisome organizer and an *E. coli* DnaC-like helicase loader, respectively (Zúñiga et al., 2002). The replication initiator (organizer) alone or together with the helicase loader is also found in the genomes of other P335-group phages often accompanied by gene encoding a single-strand DNA binding (SSB) protein (Weigel and Seitz, 2006).

The genes encoding putative replication functions of lytic *L. lactis* phages belonging to sk1-like and c2-like groups are located essentially in analogous genetic locations within the early gene expression region, upstream of the recombination genes, *ssa* (single-strand annealing) and *ssb* (single-strand binding). Our *in silico* genome analyses of sk1-like phages that were previously isolated from the Polish dairy environment (Chmielewska-Jeznach et al., 2018) indicated in this region the presence of a single gene encoding a P-loop NTPase domain. Yet, like for the majority of the early genes, the exact role of this product in DNA replication of sk1-like phages has not been experimentally studied.

P-loop NTPases hydrolyze nucleoside triphosphates (NTPs) and transfer this energy to drive various processes, such as sliding along the DNA and its unwinding (Longo et al., 2020). The structural core of P-loop NTPases is formed by a central 5-strand β-sheet surrounded by α-helices on both sides (Berger, 2008). On one rim of the sheet are localized residues taking part in NTP hydrolysis, organized in several sequence motifs characteristic for AAA+ and RecA-like proteins (for a brief summary see Berger, 2008). For instance, Walker A (also known as P-loop) and Walker B motifs together with Sensor I in AAA+ ATPases provide charged residues for NTP hydrolysis. Arginine finger completes the catalytic site *in trans* and is especially important for NTPases functioning in oligomeric assemblies, e.g., hexameric rings (Iyer et al., 2004; Miller and Enemark, 2016; Zhao et al., 2016). In the process of DNA replication, P-loop NTPases, with either AAA+ or RecA fold, play the role of clamp loaders, replication initiator proteins, helicase loaders, and DNA helicases (Davey et al., 2002; Ye et al., 2004; Miller and Enemark, 2016).

DNA helicases have key functions in DNA replication, recombination, transcription, and repair (Huttner and Hickson, 2013). The current classification divides helicases into six superfamilies (SF1-6), each characterized by unique structural and biochemical properties (Singleton et al., 2007). The distinctive features of helicases include nucleic acid-specificity (DNA or RNA), oligomerization forms (monomeric or dimeric SF1-2 and hexameric SF3-6 helicases), and unwinding directionality (5′-3′ or 3′-5′). One of the best studied models of DNA helicases is the SF4 bifunctional primase-helicase (gp4) of coliphage T7 (Richardson, 1983). It retains all sequence motifs characteristic for helicases (I, Ia, II, III, IV) and structural studies explain the detailed role of each of them for the function of this hexameric protein (Patel and Hingorani, 1993; Egelman et al., 1995; Sawaya et al., 1999; Singleton et al., 2007). Other similar helicases include bacteriophage T4 gene *41* protein (T4 gp41) and the RepA protein encoded by plasmid RSF1010–both hexameric and both preserving respective sequence motifs (Dong et al., 1995; Scherzinger et al., 1997).

In this study, we show that the product of gene *46* (GP46) of the *L. lactis* phage vB_Llc_bIBBF13 (phage F13) binds DNA as a dimer, and most likely as a higher order oligomer, and demonstrates adenosine triphosphate (ATP)-dependent 5′-3′ DNA unwinding activity. Through a series of functional experiments we show that the protein most efficiently displaces DNA strands from branched substrates, which imitate replication forks, with a 5′ extended arm. We predict that GP46_*F*13_ contains a catalytically active RecA-like protein domain homologous to RepA, RecA, DnaB, and AAA_25, among the others. Based on its sequence similarity to other helicases of known structure, e.g., RepA from *E. coli* RSF1010 plasmid and phage T7 gp4, we anticipated that GP46_*F*13_ would function as a hexameric, ring-shaped helicase. Size exclusion chromatography with multi-angle light scattering (SEC-MALS) assay indicated strong oligomerization potential of GP46, but the issue concerning its multimeric structure remains unresolved. Moreover, we identify among others an interaction of GP46_*F*13_ with host’s DnaK and DnaJ proteins as well as with single strand binding protein (SSB) and topoisomerase I (topoI). Altogether, these data suggest that GP46_*F*13_ is a DNA helicase engaged likely in replication. As *orfs* in the direct surrounding of *gp46* do not encode for known replication proteins or possess domains typical for such activity, GP46_*F*13_ is the first functionally characterized early gene product among *L. lactis* sk1-like phages that defines the putative phage replication module and for which a role as a helicase is proposed. Our findings bring us closer to unraveling the role of specific protein players in replication of virulent *L. lactis* phages in general.

## Materials and Methods

### Bacteriophage, Bacteria, and Growth Conditions

*Escherichia coli* Rosetta™ (DE3) strain (Novagen) and TG1 strain (Gibson, 1984) were grown in Luria-Bertani (LB) medium at 37°C with shaking or on LB plates supplemented with 1.5% agar. *L. lactis* IL1403 strain (Chopin et al., 1984) was grown at 30°C in M17 broth (Oxoid) supplemented with 0.5% glucose (GM17) without shaking or on GM17-agar plates. Phage F13 (GenBank accession no. MG253653) propagation was performed on *L. lactis* strain IL1403 in liquid GM17 medium supplemented with 10 mM calcium chloride (GM17-CaCl_2_) as previously described (Chmielewska-Jeznach et al., 2018).

### Cloning and Purification of GP46_*F*13_

The overproduction of the *L. lactis* phage F13 GP46 protein (GenBak accession no. ATW69819.1) was primarily attempted in *E. coli* using the IMPACT™ expression system (NEB). Yet, with this approach we were able to obtain only very low protein recovery levels, irrespectively of conditions assayed (induction temperature, IPTG and salt concentration, glycerol addition, etc.). To circumvent these problems, we cloned gene *46* (*gp46*) in the pET28a-SUMO-PIN vector (courtesy of M. Pastor). For this, *gp46* was amplified from bacteriophage F13 lysate by PCR reaction using primers SF13for/SF13rev listed in Table 1. The resultant PCR product was digested with *Bam*HI and *Xho*I (Fermentas) enzymes and ligated into *Bam*HI and *Xho*I sites of the pET28a-SUMO-PIN vector creating the N-terminal fusion plasmid for the overproduction of the GP46_*F*13_ protein (pET28:SUMO:*gp46*). Ligated molecules were transformed into the *E. coli* TG1 cells. Transformants were selected on LB-agar medium containing 50 μg/ml kanamycin at 37°C. The presence of the cloned fragment was verified by PCR using SUMOF13_F and SUMOF13_R primers (Table 1) and sequencing.

**TABLE 1:** Primers used in the study.

Primers for cloning	
SF13for	CCGC**GGATCC**ATGACTAACATATTTAATAAAGTACAG
SF13rev	GCCG**CTCGAG**TTAATCATTTTCTATTACTTTTCCTTG
SUMOF13_F	TGGGAATGGAGGAAGAAG
SUMOF13_R	CTCAAGACCCGTTTAGAG
**Primers for EMSA and helicase assays**	
ss22	Cy5-AAAAATTGAATACGCCTAAGGC
ss45	Cy5-CTCAATTCCTAAGACATCAGACAAGCCTTAGGCGTATTCAATTTT
ss68	Cy5-TATTCAGTACGGTTTTCATATACTCAATTCCTAAGACATCAGACAAGCCTTAGGCGTATTCAATTTTT
ss110	Cy5-TCACACAGGGCTACCGCTTTGCCTAACTCATTACTCGCGCCTTATTCAGTACGGTTTTCATATACTCAA TTCCTAAGACATCAGACAAGCCTTAGGCGTATTCAATTTTT
ori110_F	TCACACAGGGCTACCGCTTTGC
ori110_R	Cy5- AAAAATTGAATACGCCTAAGGC
**Primers for generating forked duplexes**	
30duplex_5′ext	Cy5-CTCAATTCCTAAGACATCAGACAAGCCTTAGGCGTATTCAATTTT
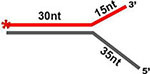	TTTTTTTTTTTTTTTTTTTTTTTTTTTTTTTTTTTTAAGGCTTGTCTGATGTCTTAGGAATTGAG
30duplex_3′ext	Cy5-CTCAATTCCTAAGACATCAGACAAGCCTTATTTTTTTTTTTTTTTTTTTTTTTTTTTTTTTTTTT
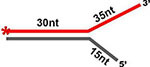	TTTTAACTTATGCGGTAAGGCTTGTCTGATGTCTTAGGAATTGAG
53duplex_5′ext	Cy5-TATTCAGTACGGTTTTCATATACTCAATTCCTAAGACATCAGACAAGCCTTAGGCGTATTCAATTTTT
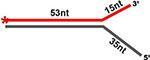	TTTTTTTTTTTTTTTTTTTTTTTTTTTTTTTTTTTCTAAGGCTTGTCTGATGTCTTAGGAATTGAGTATATGA AAACCGTACTGAATA
**Primers for generating asymmetric duplexes**	
30duplex_5′ss	TATTCAGTACGGTTTTCATATACTCAATTCCTAAGACATCAGACAAGCCTTAGGCGTATTCAATTTTT
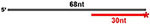	Cy5-AAAAATTGAATACGCCTAAGGCTTGTCTGA
30duplex_3′ss	TATTCAGTACGGTTTTCATATACTCAATTCCTAAGACATCAGACAAGCCTTAGGCGTATTCAATTTTT
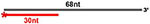	GAATTGAGTATATGAAAACCGTACTGAATA-Cy5

*Marked in bold are sites recognized by restriction enzymes used for cloning.

The recombinant plasmid pET28:SUMO:*gp46* was introduced into *E. coli* Rosetta™ (DE3) cells. Freshly grown colonies were inoculated into 1 L Luria broth (LB) medium containing 50 μg/ml kanamycin and 10 μg/ml chloramphenicol at 28°C until optical density at 600 nm (OD_600_) reached 0.7. Expression of the target protein was induced by adding isopropyl-b-D-thiogalactopyranoside (IPTG) to a final concentration of 0.4 mM and the culture was incubated further on an orbital shaker at 150 rpm for 24 h at 16°C. Next, cells were harvested by centrifugation at 5,000 rpm for 15 min at 4°C. All subsequent purification steps were carried out at 4°C. Bacterial pellet was resuspended in 30 ml of optimized suspension buffer (50 mM Tris–HCl pH 8, 500 mM NaCl, 20 mM imidazole, 2% Triton-X100) and lysed by sonication on ultrasonic cell disruptor XL (MISONIX) 10 times for 15 s with 15 s intervals on ice. Without addition of Triton-X100 the protein after sonication was in the membrane fraction. With low salt in the suspension buffer, the protein recovery was very low. After sonication, the cell debris was then removed by centrifugation at 11,000 rpm for 1 h at 4°C. The supernatant was applied on a HisTrap FF crude column pre-packed with the Ni-Sepharose 6 Fast Flow affinity medium (GE Healthcare) and equilibrated with column buffer (50 mM Tris–HCl pH 8, 500 mM NaCl, 20 mM imidazole). The resin-bound protein was rinsed with 50 ml of column buffer and washed out with elution buffer (50 mM Tris–HCl pH 8, 500 mM NaCl, 300 mM imidazole). The eluate was digested by SUMO protease at 4°C overnight to remove SUMO-tag and then analyzed for the presence of GP46_*F*13_ by SDS-PAGE. Protein concentration was determined using the Bradford assay on a UV spectrophotometer (Shimadzu).

### Ion Exchange Chromatography

Further protein purification was performed by FPLC (Fast Protein Liquid Chromatography) on the ÄKTA Purifier using the HiTrap^®^ Heparin HP column (GE Healthcare) equilibrated with 50 mM Tris-HCl buffer (pH 8). Before applying on the column, the elution buffer was exchanged to a buffer containing 50 mM Tris–HCl pH 8, 100 mM NaCl, by dialysis. The GP46_*F*13_ protein sample was loaded on the column at a flow rate of 0.4 ml/min. Protein elution was carried out using a growing gradient concentration of NaCl (from 100 mM to 1 M) in 50 mM Tris–HCl pH 8 buffer. The peak fractions were analyzed by SDS-PAGE and stored after adding 50% glycerol (v/v) at −20°C for further experiments.

### DNA Binding Assays

The DNA-binding activity of GP46_*F*13_ was evaluated by electrophoretic mobility-shift assay (EMSA) with fluorescently labeled oligonucleotides (Cy5 on 5′ end) differing in size (22, 45, 68, 110 nt). The dsDNA 110 nt substrate was prepared by PCR reaction using *L. lactis* c2 phage genome as a template and a pair of complementary primers (ori110_F/ori110_R). The binding reactions were carried out for 30 min at 30°C in binding buffer (20 mM Tris–HCl pH 8, 50 mM NaCl, 1 mM DTT, 1 mM EDTA, 0.1 mg/ml of BSA, 2.5% glycerol) in 20 μl reaction volumes containing 1 nM of appropriate DNA substrate and the indicated amounts of analyzed protein (0.1–0.75 μM). Reaction samples were loaded onto 7% non-denaturing polyacrylamide gels and resolved for 40 min at 100 V in 0.5 × TBE buffer at 4°C with 1-h pre-run. Gels were developed using FluorChemQ MultiImageIII ChemiImager (Alpha Innotech, San Leandro, CA, United States) and the images were captured using Alpha View software (Alpha Innotech, San Leandro, CA, United States).

### Helicase Activity Assay

Helicase activity of the GP46_*F*13_ protein was assessed on a variety of DNA substrates (Table 1). Asymmetric or forked DNA duplexes were prepared by annealing a 5′ Cy5-labeled oligonucleotides to a series of partially complementary unlabeled ssDNAs (as described by Curti et al., 2007). The helicase activity was assayed by mixing the protein with 1.5 nM of Cy5-labeled DNA substrate in a standard reaction buffer containing (if not stated otherwise) 50 mM Tris-HCl, pH 8, 50 mM NaCl, 5 mM MgCl_2_ at 20 μl of final volume. Reactions were initiated by the addition of 5 mM ATP (or other NTP/dNTP), and the mixtures were incubated at 30°C for 30 min (if not otherwise stated). Reactions were terminated by the addition of 6 μl of stop solution (10% glycerol, 0.4% SDS, 50 mM EDTA). To minimize reannealing of the unwound oligonucleotides, a 10-fold molar excess of unlabeled DNA trap, corresponding to the Cy5-labeled strand was added after 10 min of the reaction. As a negative control, the substrates were incubated in the reaction mix in the absence of the protein. Samples were resolved in a 15% polyacrylamide gel in 0.5 × TBE buffer for 40 min at 18°C with a 30-min pre-run. For data analysis, gels were developed in FluorChemQ MultiImageIII ChemiImager (Alpha Innotech, San Leandro, CA, United States) and the images were captured using Alpha View software (Alpha Innotech, San Leandro, CA, United States). The unwound DNA was quantified using ImageJ (Image Processing and Analyzing In Java).

### Size Exclusion Chromatography With Multi-Angle Light Scattering Analysis

Size exclusion chromatography (SEC) coupled with multi-angle light-scattering (MALS) was done using a high-performance liquid chromatography (HPLC) equipment (1260 Infinity LC, Agilent Technologies Inc., Santa Clara, CA, United States) connected to a UV detector, a MALS detector (DAWN HELEOS II, Wyatt Technology Santa Barbara, CA, United States), and a differential refractometer (Optilab T-rEX, Wyatt Technology, Santa Barbara, CA, United States). The GP46_*F*13_ sample (100 μl) purified as described earlier was injected at 1.5 mg/ml final concentration onto a Superdex 200 Increase 10/300 column (GE Healthcare, Milwaukee, WI, United States) and run at room temperature and a 0.5 ml/min flow rate in buffer containing 50 mM Tris–HCl pH 8, 500 mM NaCl, 20 mM imidazole. Output data were analyzed using ASTRA v. 6 software (Wyatt Technology, Santa Barbara, CA, United States).

### Pull-Down Assay

#### Preparation of Phage-Infected Bacterial Culture Extracts

The *L. lactis* IL1403 host strain culture (160 ml) was grown to OD_600_ 0.4. After that a portion of the culture (40 ml) was taken, centrifuged (6,000 rpm, 4°C, 2 min) and frozen in liquid nitrogen (negative control). The remaining part was supplemented with 10 mM CaCl_2_ and infected with *L. lactis* phage F13 at MOI of 5. The infected cell culture was further incubated at 30°C and 40 ml portions were taken at times 5, 10, and 15 min after infection, centrifuged (6,000 rpm, 4°C, 2 min) and frozen in liquid nitrogen to stop the phage cycle and stored at −80°C until further use. At the time of analysis, the phage-infected culture samples were thawed. The pellet was resuspended in phosphate buffered saline (pH 7.4), bacterial cells were broken using glass beads (0.10–0.11 mm) and centrifuged (13,000 rpm, 4°C, 30 min). The supernatant samples containing phage-infected bacterial cell extracts were used in further analyses.

#### Immobilization of the GP46_*F*13_ Protein on the Ni^2+^ Resin

The GP46_*F*13_-His tagged protein from 250 ml culture was immobilized in 1 ml of HisPur™ Ni-NTA Superflow Agarose (Thermo Scientific™) applied to 5-ml PP columns (GE Healthcare) and rinsed with column buffer (50 mM Tris–HCl pH 8, 500 mM NaCl, 20 mM imidazole). Then, crude cell lysates of IL1403 host cells at 0, 5, 10, 15 min after infection with phage F13 were added and incubated overnight at 4°C. After excessive washing with 100 volumes of column buffer, the protein content bound to the resin was analyzed using mass spectrometry in the Mass Spectrometry Lab at IBB PAS.

#### Mass Spectrometry Analysis

The assay was performed with the use of the nanoHPLC nanoACQUITY system and the C18 pre-column and nanoAcquity BEH C18 column. Mobile phase consisted of water supplemented with 0.1% formic acid (FA) and acetonitrile with gradient elution. The column out-put was directly connected with the Q Exactive spectrophotometer. Fragmentation was done using the HCD method. Preprocessing and protein identification was performed respectively by employing the Mascot Distiller (version 2.6) and Server (version 2.4.1) and the *L. lactis* IL1403 (GenBank accession no.: AE005176.1) and phage F13 (GenBank accession no.: MG253653.1) protein sets. In order to distinguish non-specific binding to the resin, the obtained hit results were normalized against control affinity resin without tagged GP46_*F*13_.

### Bioinformatic Analyses

Homologs of the GP46_*F*13_ protein were collected using PSI-BLAST (Altschul et al., 1997) search (three iterations, inclusion threshold 0.05) against non-redundant (nr) database with GP46_*F*13_ protein sequence (GenBank accession no. ATW69819.1) as a query. Sequence similarity to proteins with known structures and other Pfam families was scanned with HHpred at MPI Bioinformatics Toolkit (Zimmermann et al., 2018). All multiple sequence alignments were calculated using Mafft (L-INS-i) (Katoh et al., 2002). GP46_*F*13_ structure modeling was done using AlphaFold2 (Jumper et al., 2021). Multiple structure alignments between identified homologs of known structure and GP46_*F*13_ model were prepared manually.

Sequences of proteins belonging to other helicase families identified by HHpred were collected using PSI-BLAST searches starting with one representative sequence for each family. All the sequences, including GP46_*F*13_ homologs, were altogether clustered to 65% of sequence identity using CD-HIT (Li et al., 2001) to reduce redundancy. The dataset was further clustered using CLANS (Frickey and Lupas, 2004).

## Results

### *In silico* Analyses of GP46_*F*13_ Amino Acid Sequence and 3-D Fold

**FIGURE 1:**
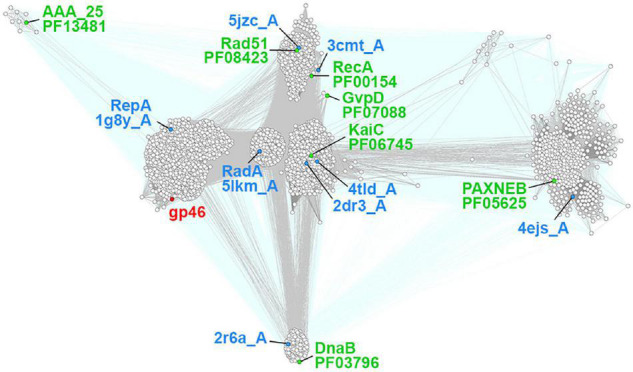
Sequence clustering of GP46_*F13*_ and the closest protein families. Each point represents one protein sequence. Protein sequences used as queries for collecting family members are marked with green dots and sequences of proteins of known structures with blue ones. Lines representing mappings with *P*-values lower and higher than 1E-15 are drawn in gray and teal, respectively.

**FIGURE 2:**
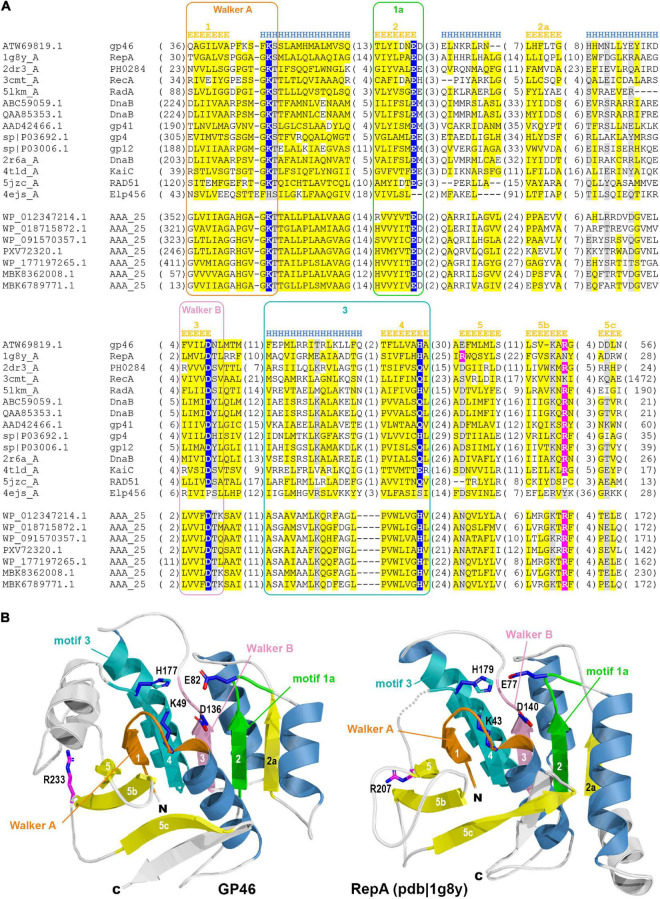
**(A)** Multiple sequence alignment of GP46_*F13*_ and the representatives of related families. Sequences are labeled with GenBank accession number, Uniprot ID, or PDB code. Sequence conservation is denoted with yellow and gray highlighting for non-polar and charged or polar residues, respectively. The numbers of residues omitted in the alignment are provided in parentheses. Residues involved in NTP hydrolysis are highlighted with blue, and the arginine from arginine finger–in pink. Sequence motifs are outlined with rectangles. Secondary structure elements are labeled according to James M Berger’s summary on helicase structures (Berger, 2008). **(B)** Comparison between 3D model of GP46_*F13*_ (left) and structure of the RepA (pdb| 1g8y). Non-core structural elements not present in the alignment **(A)** are rendered in white. Structural elements corresponding to defined motifs are colored in respective colors.

The GP46 protein of *L. lactis* phage F13 (GP46_*F*13_) according to HHpred mappings displays high sequence similarity (all hits with scores > 90 and estimated probability of being true positive > 99%) to several P-loop NTPase protein families, including RepA, RadA, RecA, GvpD, DnaB, RAD51, KaiC, and AAA_25 (Figure 1 and Supplementary Table 1). RepA and DnaB are replicative helicases (Niedenzu et al., 2001; Bailey et al., 2007), RadA is a DnaB-type helicase interacting with RecA in homologous recombination (Marie et al., 2017), RAD51 and RecA are recombinases forming helical filaments seeking for homology in homologous recombination (Chen et al., 2008; Short et al., 2016), GvpD regulates gas vesicle formation in Archaea, KaiC is an ATPase controlling cyanobacterial circadian clock (Abe et al., 2015) and AAA_25 proteins have no known function described yet. Due to the functional diversity of homologous protein families and taking into consideration sequence and structural similarities between them we performed a more detailed sequence clustering in order to provide a more reliable classification and annotation. Obtained results suggest that GP46_*F*13_ is closely related to the RepA family. It retains all sequence motifs characteristic for NTP hydrolysis, including residues Lys49 from Walker A (P-loop) motif, Glu82 (motif 1a), Asp136 (Walker B), and His177 (motif 3) (Figure 2A). It also has an arginine finger (Arg233) which might suggest a similar catalytic mechanism. Interestingly, the AAA_25 family identified among GP46_*F*13_ homologs also displays RecA-like features and will probably not function as an AAA ATPase (Figure 2A).

Molecular modeling of GP46_*F*13_ confirmed conservation of all structural elements essential for helicase function. The model computed by AlphaFold2 (pLDDT score 83.9; scores above 70 are regarded as confident) demonstrates no major differences compared to the RepA structure (Figure 2B) which suggest that GP46_*F*13_ may also function as hexameric helicase. Despite the similarity of GP46_*F*13_ to RepA, it retained the arginine finger in its canonical position at strand 5b, while in RepA it is located on the proximal strand 5 from where it executes its catalytic function.

### Cloning and Purification the GP46_*F*13_ Protein

To obtain sufficient quantities of GP46_*F*13_ protein for *in vitro* studies the pET28a:SUMO-PIN expression system was used. For this, the phage F13 *gp46* open reading frame encoding the 298-AA product was amplified by PCR and fused at its N-terminal end with 6xHis tag and SUMO protein-encoding sequences of the pET28a:SUMO-PIN vector in *E. coli*. The GP46_*F*13_ 6xHis- and SUMO-tagged protein was purified by nickel-affinity chromatography, and after removal of the SUMO tag, further purification was done using the heparin-sepharose column (Supplementary Figure 1). SDS-PAGE analysis of the final purification protein revealed a major protein band with a molecular weight of approximately 34 kDa, which corresponds to the molecular weight calculated from the amino acid sequence (Supplementary Figure 1).

### GP46_*F*13_ Binding to DNA Substrates

In order to elucidate the binding of GP46_*F*13_ to different DNA substrates *in vitro* EMSA assays were performed. For this purpose 1 nM of 5′ fluorescently labeled DNA substrates (Table 1) were incubated with varying GP46_*F*13_ protein concentrations.

**FIGURE 3:**
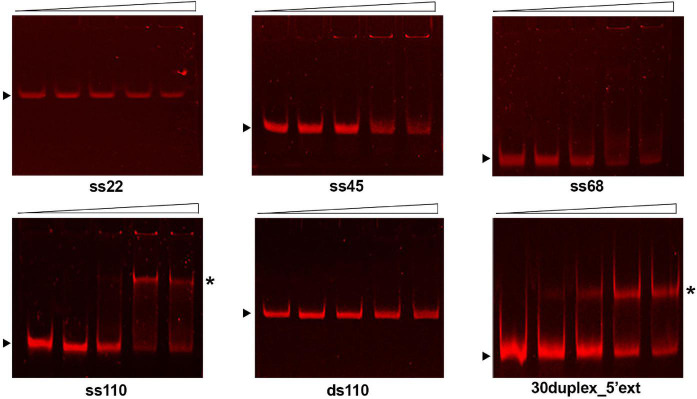
Binding of the GP46_*F13*_ protein to DNA substrates. Rising concentrations of GP46_*F13*_ (0.1, 0.25, 0.5, and 0.75 μM) were incubated with 1 nM of ssDNA (ss22, ss45, ss68, ss110), dsDNA (110ds) or forked substrate with elongated 5′ tail (30duplex_5′ext) for 30 min at 30°C in binding buffer (20 mM Tris–HCl pH 8, 50 mM NaCl, 1 mM DTT, 1 mM EDTA, 0.1 mg/ml of BSA, 2.5% glycerol) in 20 μl reaction volume. Reaction samples were analyzed on 7% native polyacrylamide gels. Nucleoprotein complexes are indicated by asterisk and unbound DNA substrates by black arrow.

As shown in Figure 3, GP46_*F*13_ interacts with ssDNA substrates of different length (22, 45, 68, and 110 nt) with nanomolar affinity and the relative amounts of unshifted ssDNA decrease proportionally to protein concentrations added. Clear retardation complexes could be seen for the 110-nt ssDNA (ss110). For shorter (ss22, ss45, ss68) substrates, a formation of discrete protein-DNA aggregates in the wells of the gel were observed. Although we see a fraction of the ssDNA substrate stuck in the well, most probably associated with the tendency of the protein to aggregate *in vitro*, our results indicate that GP46_*F*13_ can interact with DNA. At the same time, GP46_*F*13_ did not bind to dsDNA; no nucleoprotein complexes were observed with the 110-bp dsDNA (ds110) vs. ss110 substrate (Figure 3). To examine whether GP46_*F*13_ has affinity for substrates mimicking the replication forks, we performed EMSA assays using a branched 30-bp duplex with a short 15-nt 3′ tail and an extended, 35-nt 5′ ssDNA overhang (30duplex_5′ext). We show that the GP46_*F13*_ gives clear protein-DNA complexes with 30duplex_5’ext, suggesting that GP46_*F*13_ can bind also to heteroduplex structures.

### DNA Unwinding by GP46_*F*13_

**FIGURE 4:**
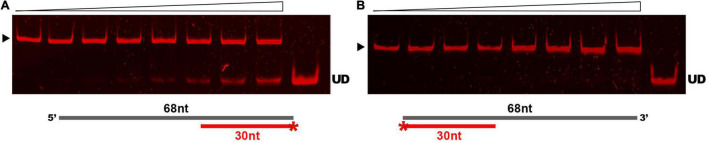
Substrate-specific unwinding by GP46_*F13*_. Reactions with asymmetric DNA substrate **(A)** 30duplex_5’ss or **(B)** 30duplex_3’ss. Rising concentrations of the GP46_*F13*_ protein (0, 0.1, 0.25, 0.5, 0.75, 1.0, 1.5, 2.0 μM) was incubated with DNA substrate (1.5 nM) for 30 min at 30°C in reaction buffer (50 mM Tris-HCl, pH 8, 50 mM NaCl, 5 mM MgCl_2_) and 5 mM ATP. Reaction samples were analyzed on native 15% polyacrylamide gels. ▶ indicates the expected migration of the substrate; UD indicates the expected migration of the unwound DNA (Cy5-labeled oligonucleotide) marked on the scheme in red and with an asterisk.

Results of our comparative analyses *in silico* suggested that GP46_*F*13_ is conserved in key amino acids and has similar fold structure to DNA helicases, including RepA from plasmid RSF1010 and phage T7 gp4 protein. To evaluate whether GP46_*F*13_ exhibits also a similar DNA unwinding ability, we performed helicase activity assays under various conditions. For these we used a series of asymmetric substrates with an ssDNA overhang from either 5′ or 3′ end or forked substrates with two free ssDNA ends of different length (Table 1). We determined that the GP46_*F*13_ protein was not active on blunt-ended dsDNA substrates (results not shown). Instead, we observed a preference for an asymmetric DNA substrate with 5′ tail (30duplex_5′ss), but not with a 3′ overhang (30duplex_3′ss), indicative of a 5′–3′ directionality of the protein’s unwinding activity (Figure 4).

**FIGURE 5:**
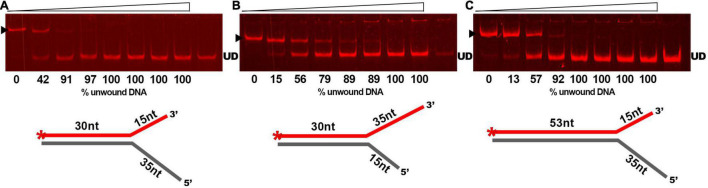
Substrate specific unwinding by GP46_*F13*_. Reactions with forked DNA substrates: **(A)** 30duplex_5′ext; **(B)** 30duplex_3′ext; **(C)** 53duplex_5′ext. Rising concentrations of the GP46_*F13*_ protein (0, 0.1, 0.25, 0.5, 0.75, 1,0, 1.5, 2.0 μM) were incubated with specific branched substrate (1.5 nM) for 30 min at 30°C in reaction buffer (50 mM Tris-HCl, pH 8, 50 mM NaCl, 5 mM MgCl_2_) and 5 mM ATP. Reaction samples were analyzed on native 15% polyacrylamide gels. ▶ indicates the expected migration of the substrate; UD indicates the expected migration of the unwound DNA (Cy5-labeled oligonucleotide) marked on the scheme in red and with an asterisk.

As DNA helicases can act at replication forks by breaking down the hydrogen bonds and separating the strands of the duplex DNA, we examined whether GP46_*F*13_ is capable of unwinding branched duplexes that mimic such structure. For this we used three types of substrates varying in the length of the dsDNA region and the free 3′ and 5′ ss ends (Table 1). Results showed clearly that extension (from 15 nt to 35 nt) of the 5′ end increases the rate of the separation of duplex DNA strands (Figures 5A–C). A 15-nt long 5′ ssDNA overhang was sufficient enough for GP46_*F*13_ unwinding of the forked substrate. We also showed that the protein is capable of displacing longer (53 bp) stretches of duplex DNA with 5′ tail (Figure 5C). Yet, liberation of the labeled oligonucleotide product occurred at slightly higher protein:DNA molar ratio for the 53duplex_5′ext vs. 30duplex_5′ext substrate (Figures 5A,C). To this end, the 30duplex_5′ext was recognized as the most preferable substrate and was used in further assays.

**FIGURE 6:**
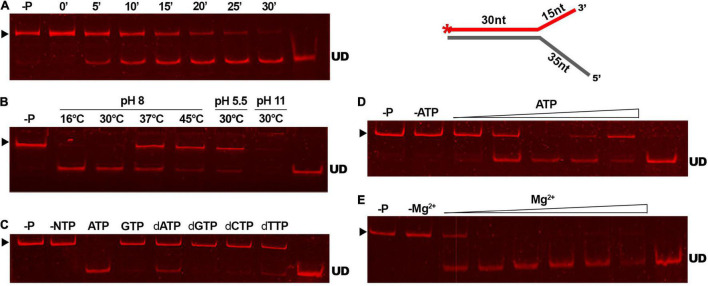
The unwinding activity of GP46_*F13*_ under different reaction conditions. The GP46_*F13*_ protein (0.5 μM) and Cy5-labeled branched substrate (1.5 nM) with extended 5′ tail (30duplex_5′ext) were incubated under varying conditions: time (0, 5, 10, 15, 20, 25, 30 min) **(A)**; temperature (16°C, 30°C, 37°C, 45°C) and pH (5.5, 8.0, 11) **(B)**; NTPs or dNTPs (5 mM) **(C)**; ATP (0, 1, 2, 5, 10, 15 mM) **(D)**; and MgCl_2_ (0, 1, 2, 5, 10, 15, 20 mM) **(E)**. Reaction samples were analyzed on native 15% polyacrylamide gels. –P; control reaction without protein. –NTP/–ATP; control reaction without NTPs/ATP. –Mg^2+^; control reactions without Mg^2+^. ▶ indicates the expected migration of the substrate; UD indicates the expected migration of the unwound DNA (Cy5-labeled oligonucleotide) marked on the scheme in red and with an asterisk.

To determine the efficiency of the reaction of GP46_*F*13_ on the substrate DNA, we conducted a time-dependent DNA unwinding assay (Figure 6A). Results showed that the extent of displaced oligonucleotide increased in time and after 30 min it was almost completely liberated from the duplex DNA. Optimal temperature and pH conditions for DNA unwinding were determined to be at 30°C and pH 8 (Figure 6B). Under these conditions the strands of the duplex DNA were completely separated. As the unwinding activity of DNA helicase proteins is known to be fueled by various nucleotide substrates, we tested the specific requirement of GP46_*F*13_ for NTPs or dNTPs (Figure 6C). Results of the assay demonstrated efficient separation of duplex strands in a 30-min reaction in the presence of 5 mM ATP. Moderate displacement of the labeled oligonucleotide was detected for dATP and dTTP at the same concentrations, while no unwinding activity was observed for the remaining nucleotide substrates. We thus concluded that the unwinding activity of GP46_*F*13_ is ATP-dependent, while the binding to DNA can occur in the absence of ATP. Next, we examined the influence of rising ATP concentrations on the unwinding activity of GP46_*F*13_ (Figure 6D). Complete unwinding of the forked DNA substrate was observed at 5 mM ATP under the tested conditions, indicating that this is the optimal concentration of the supplied nucleotides. Lower and higher ATP concentrations allowed only for partial separation of duplex DNA strands. The activity of ATP-dependent helicases relies strongly on the presence of metal cofactors, particularly Mg^2+^ ions. The ratio of ATP to Mg^2+^ was documented previously to influence the efficiency of helicase activity. Therefore, we also investigated the effects of different concentrations of Mg^2+^ under constant ATP level on the unwinding activity of GP46_*F*13_. The most efficient separation of duplex DNA was determined when Mg^2+^ and ATP were supplied at an equimolar (5 mM) concentration (Figure 6E).

### GP46_*F*13_ Multimerizes in Solution

**FIGURE 7:**
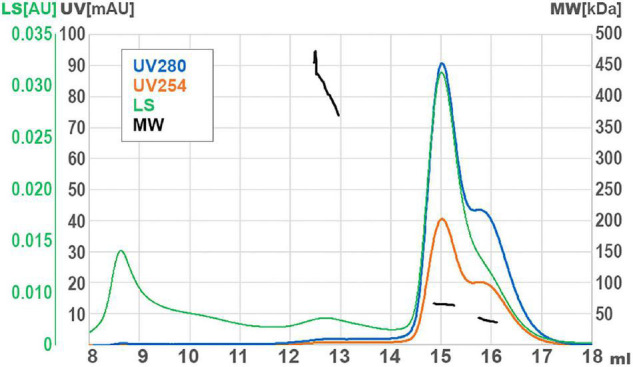
Size exclusion chromatography (SEC) with multi-angle light scattering (MALS) analysis for GP46_*F13*_. UV absorption and light scattering (LS) are shown on the left axis, molecular weight of protein (MW) are on the right axis. Short black lines mark the detected molecular mass.

DNA helicases generally function as multimeric forms (dimers or hexamers) (West, 1996). In order to infer the oligomerization status of GP46_*F*13_, we performed SEC-MALS analysis. For purification of GP46_*F*13_, we used in all the buffers high salt concentration (500 mM NaCl) to increase solubility and stability of the protein (see Section “Materials and Methods”). In turn, formation of oligomeric forms is generally promoted at low salt concentrations (50–200 mM). Thus, we initially performed several SEC runs at low salt conditions (50 and 200 mM). Unfortunately, in these cases the protein was eluted as a large multimeric form in the void volume of the SEC column (Supplementary Figure 2). To stabilize the protein, we increased the salt concentration to 500 mM and obtained a major single peak. By DLS assays we checked that addition of cofactors (DNA, ATP, and Mg^2+^) does not influence GP46_*F*13_ oligomerization (data not shown). In the light of these data, SEC-MALS analysis was performed at restored higher salt conditions without cofactors and revealed several GP46_*F*13_ populations–a monomer and two oligomeric forms (Figure 7). The main fraction had a mass value of 65 kDa, the second peak corresponded to 39 kDa fraction, while the smallest peak was dispersed with a mean mass of 410 kDa; yet, the signal detecting the largest peak was too weak for accurate size determination. The values detected were similar to the theoretical molecular weight (MW) of a dimer (2 × 34.25 = 68.5 kDa), a monomer (34.25 kDa), and possibly a 12-mer (12 × 34.25 = 411 kDa). Results of the assay indicate that GP46_*F*13_ can self-associate in solution. The presence of the monomeric fraction indicates that under the tested conditions the multimeric protein structure is not stable since high salt concentrations prompt possible dissociation. On the other hand, it is surprising to observe multimeric forms in a buffer containing 500 mM NaCl. This proves a strong GP46_*F*13_ tendency for oligomerization and suggests assembly of GP46_*F*13_ into higher multimeric forms.

### Interaction of GP46_*F*13_ With Proteins From Phage F13 and the *Lactococcus lactis* IL1403 Host Strain

To probe for potential phage or host protein partners, we performed pull-down assays with tagged GP46_*F*13_ as bait, followed by LC-MS and computational analysis. We detected high distribution of peptides corresponding to GP46_*F*13_ and to three other non-structural phage proteins, i.e., GP23 (GenBank accession no.: ATW69796.1), GP28 (GenBank accession no.: ATW69801.1), and GP32 (GenBank accession no.: ATW69805.1), suggesting their interaction with the affinity-bound GP46_*F*13_ (Supplementary Table 2). By BlastP and HHPred searches we found that GP28 carries a Zn^2+^ binding domain, GP32 contains a conserved DUF3310 (domain of unknown function), and GP23 has no significant hits. Thus, the explicit function of all three detected proteins and their role in phage replication remains hypothetical. By LC-MS we also detected significant amounts of peptides representing host *L. lactis* IL1403 proteins—DnaK (GenBank accession no.: AAK05052.1), DnaJ (GenBank accession no.: AAK06322.1), topoisomerase I (GenBank accession no.: AAK05328.1), and SSB (GenBank accession no.: AAK06288.1). All of these proteins have previously been shown to be engaged in DNA replication. Affinity interactions of these proteins with the tagged GP46_*F*13_ in our pull down assay are a strong suggestion that they are partners in a common biological process. Their specific interaction with GP46_*F*13_ demands further experimental confirmation.

## Discussion

It is generally considered that in virulent *L. lactis* sk1- and c2-like phages functions involved in phage DNA replication, recombination and translation are encoded in the early region. Yet, the majority of the genes therein remain uncharacterized. This hinders our understanding of the early stage of infection by *L. lactis* phages. We examined the activity of an early gene product, GP46, encoded by a virulent sk1-like *L. lactis* phage F13. By gene order, *gp46* is located as the third open reading frame in the phage early-transcribed gene region. Its immediate genomic neighbors encode proteins of hypothetical function and the precise role of this region in phage development remains unknown. We demonstrate that GP46_*F*13_ belongs to the RepA cluster of P-loop NTPases, which are involved in managing diverse DNA processing pathways, such as DNA replication, repair, and recombination (Iyer et al., 2004; Miller and Enemark, 2016). It possesses all features characteristic for known hexameric helicases. For instance, it retains a complete catalytic site (Lys49, Glu82, Asp136, and His177) complemented with an arginine finger (Arg233), typical for oligomeric helicases.

GP46_*F*13_ is also highly similar to proteins annotated as hypothetical DNA polymerases encoded in homologous genome regions in other lytic phages. Yet, none of them seems to be functionally characterized. Several studies describe the identification of the origin of replication of sk1-like phage genomes (Chandry et al., 1997; Crutz-Le Coq et al., 2002), but no specific protein functions associated with phage DNA replication have been identified at that time. By employing a series of experimental approaches, including protein structure analysis, DNA binding and unwinding assays, and investigation of protein-protein interactions, we examined the activity of GP46_*F*13_. Our study is the first to functionally characterize a DNA helicase function encoded in the early gene region of a virulent *L. lactis* phage and propose its role in phage replication.

The closest structurally characterized homolog of GP46_*F*13_, RepA, from a broad-host range plasmid RFS1010, is an ATP-dependent hexameric 5′-3′ replicative helicase (Scherzinger et al., 1997). Our results demonstrate that in many aspects GP46_*F*13_ remains similar to RepA_*RSF*1010_, including DNA binding, dissection of forked substrates in the 5′-3′ direction in the presence of ATP and the ability to form oligomers. Since substrate recognition by helicases engaged in DNA replication is rather structure- than sequence-specific (Anand and Khan, 2004; George et al., 2009), the effective assembly on branched duplex DNAs mimicking the replication forks implies the preference of the protein to unwind these types of substrates. RepA_*RSF*1010_ is known to have the highest affinity to forked substrates but also to asymmetric DNA duplexes with free 5′ ends (Scherzinger et al., 1997). GP46_*F*13_ demonstrates a similar preference–unwinding of the DNA duplexes (both branched and asymmetric) was promoted given a free 5′ tail was provided. Interestingly, the GP46_*F*13_ showed more robust unwinding activity on the replication fork-mimicking substrate with extended 5′ end and a short 3′ flap than on DNA with only one free (5′) tail. These observations further support our hypothesis that GP46_*F*13_ plays a role in DNA processing pathway(s).

The DNA unwinding activity of helicases is propelled by the presence of nucleoside triphosphates (NTPs) and ion cofactors (Tuteja and Tuteja, 2006). Energy deriving from the hydrolysis of specific NTPs (or dNTPs) is the general driving force of helicase-based separation of DNA strands. Other studies showed that the activity of different DNA helicases is based on specific nucleotide requirements (Lohman and Bjornson, 1996). In our experiments the unwinding activity of GP46_*F*13_ was most potent in the presence of ATP and Mg^2+^ at equimolar ratio. For other NTPs/dNTPs and in the absence of Mg^2+^ lower rates or no strand displacement was observed. In this respect GP46_*F*13_ also resembles the RepA_*RSF*1010_ which exhibited its highest activity in the presence of ATP and Mg^2+^ (Scherzinger et al., 1997).

Replicative DNA helicases act as ring oligomers that catalyze unwinding of dsDNA at replication forks (Patel and Picha, 2000; Trakselis, 2016). In general, various P-loop NTPases exhibiting helicase activity were determined to form oligomeric structures, including the bovine papillomavirus E1 and the Large-T-antigen of the simian virus 40 (Hickman and Dyda, 2005). Results of our SEC-MALS experiments showed that GP46_*F*13_ forms oligomers in solution, indicating that it most probably operates in such form, rather than as a monomer. Helicases work to unwind duplex DNA to generate ssDNA stretches that serve as intermediates in various DNA metabolic processes, including replication, recombination, or repair. In the cell, DNA helicases work as part of complex machinery systems which they form with other proteins (Patel and Picha, 2000). Moreover, the loading of helicases onto DNA is facilitated by the interaction with specific proteins at sequence-specific sites (Konieczny, 2003). For example, the RepA of plasmid RSF1010 is loaded at the oriV (origin of vegetative DNA replication) of plasmid RSF1010 initially melted by the plasmid-encoded RepC protein (Scherzinger et al., 1991). Replication of RSF1010 *in vitro* was shown to require chromosomally encoded proteins, including *E. coli* DNA gyrase, DnaZ protein (gamma subunit of PolIII holoenzyme), and SSB. For phage T7 gp4 primase-helicase, interactions were detected with phage-encoded gp2.5 SSB and gp5 DNA polymerase protein as well as with the host (*E. coli*) processivity factor (i.e., thioredoxin) (Zhang et al., 2011). The G40P helicase of *B. subtilis* phage SPP1 efficiently assembles on the DNA in the presence of phage G38P and G39P proteins (Ayora et al., 1999). To determine what are the protein partners of GP46_*F*13_ from both the phage and host side, we performed LC-MS analysis. Results of the assay revealed, among others, self-interaction of GP46_*F*13_ as well as interaction with host proteins, DnaK/DnaJ, SSB, and topoI. DnaK/DnaJ proteins set up a known chaperone protein system, both in Gram(−) and Gram(+) bacteria. They have previously been shown to be involved in replication of plasmids R1, P1, and F (Żylicz et al., 1989; Wickner et al., 1991; Giraldo-Suárez et al., 1993). In phage λ, DnaK/DnaJ were found to destabilize the oriλ:O:P:DnaB complex and release of the DnaB helicase from λP, which concomitantly led to the stimulation of its unwinding activity. Interaction of DnaB-like DNA helicases with SSB was also reported for several bacterial species. In *E. coli*, the native SSB protein (but also other heterologous SSBs) was recognized to stimulate the activity of the cognate DnaB helicase, albeit direct interaction of these two proteins is under discussion (Biswas et al., 2002). In turn, the SSB protein of phage T7, gp2.5, also physically interacts with its cognate helicase-primase (gp4) and by this modulates its DNA-unwinding activity (He and Richardson, 2004; Marintcheva et al., 2006). Finally, multiple evidence of the role of topoI in replication cannot preclude the possible contact with DNA helicases. Studies in *E. coli* have shown that the negative supercoiling relaxation by topoI at oriC (origin of replication) sites makes this region accessible for the replication machinery to initiate replication (Kraemer et al., 2019; Brochu et al., 2020). It has also been proposed that topoI may slow down DnaB helicase progression and halt replication by preventing excessive negative supercoiling at chromosomally encoded termination regions (Ters) (Valjavec-Gratian et al., 2005). Yet, confirmation of the direct interaction of all above-mentioned proteins with GP46_*F*13_ necessitates further studies.

Our study provides experimental evidence that GP46_*F*13_ is a 5′-3′ helicase. So far it is the only identified and functionally characterized protein with DNA unwinding activity encoded in the early gene region among sk1-like lytic *L. lactis* phages. The genomic localization and identified activity of GP46_*F*13_ implies its role in DNA replication and we suspect it defines the putative replication module. Weigel and Seitz (2006) have recognized a range of distinct replication modules of phage infecting various hosts. Yet, the study describes only the replication modules of *L. lactis* temperate P335-like phages. Results of this study brings us closer to deciphering the “dark matter” of the early gene expression region in sk1-like phages and other lytic lactococcal phages in general as well as the protein players engaged in the replication process of this group of phages.

## Data Availability Statement

The original contributions presented in the study are included in the article/Supplementary Material, further inquiries can be directed to the corresponding author.

## Author Contributions

MC-J performed the experimental investigations, prepared the tables, and edited the manuscript. KS performed the bioinformatics analyses, prepared figures and tables, wrote and edited sections of the manuscript. KK performed the SEC-MALS analysis and edited parts of the manuscript. JB co-supervised the work. AS co-supervised the work, responsible for the experimental concept, critically reviewed and analyzed all data, and wrote the main text of the manuscript. All authors have read, revised, and approved the manuscript.

## Conflict of Interest

The authors declare that the research was conducted in the absence of any commercial or financial relationships that could be construed as a potential conflict of interest.

## Publisher’s Note

All claims expressed in this article are solely those of the authors and do not necessarily represent those of their affiliated organizations, or those of the publisher, the editors and the reviewers. Any product that may be evaluated in this article, or claim that may be made by its manufacturer, is not guaranteed or endorsed by the publisher.

## Supplementary Material

Supplementary Figure 1 GP46_F13_ protein overproduction and purification steps.

Supplementary Figure 2 SEC analysis of GP46_F13_ protein fractions at different salt concentrations.

Supplementary Table 1 Protein data set of GP46_F13_ homologs and related protein families.

Supplementary Table 2 Mass spectrometry results presented as specific peptide hits against *Lactococcus lactis* IL1403 (GenBank accession no.: AE005176.1) and phage F13 (GenBank accession no.: MG253653.1) protein sets.
