# Supplementary material for: An Adenosine Triphosphate- Dependent 5′-3′ DNA Helicase From sk1-Like Lactococcus lactis F13 Phage
Source: Front Microbiol. 2022 Mar 15;13:840219. doi: 10.3389/fmicb.2022.840219 (PMC8965321; doi:10.3389/fmicb.2022.840219)
Supplement: Supplementary Figure 1 — GP46F13 protein overproduction and purification steps. [file Data_Sheet_1.pdf]

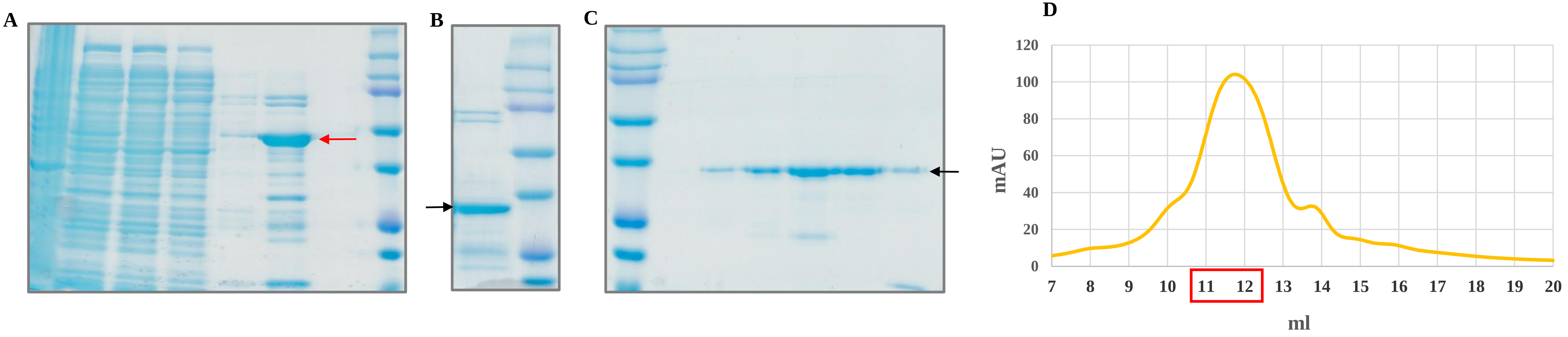

Supplementary Figure 1. GP46$_{F13}$ protein overproduction and purification steps.
(A) lanes 1: sonicate pellet fraction; 2: sonicate soluble fraction; 3: Ni^{2+} column flowthrough; 4: elution fraction with low imidazole concentration (20 mM); 5-7: elution fractions with high imidazole concentration (300 mM); 8: molecular size protein marker (Precision Plus Protein™ Dual Color Standards, BioRad); **(B)** lanes 1: GP46$_{F13}$ after removal of SUMO tag and dialysis (in 50 mM Tris-HCl pH 8, 100 mM NaCl); 2: molecular size protein marker. SDS-PAGE gel **(C)** and FPLC chromatogram **(D)** of GP46$_{F13}$ fractions eluted from the HiTrap® Heparin HP column. **(C)** Lanes 1: molecular size protein marker; 2-7: studied GP46$_{F13}$ fractions 11-12 ml boxed in red on the chromatogram **(D)**. Position of GP46$_{F13}$ (~34 kDa) is indicated by the black arrow; red arrow indicates the fusion GP46$_{F13}$ -SUMO protein band (~51 kDa).

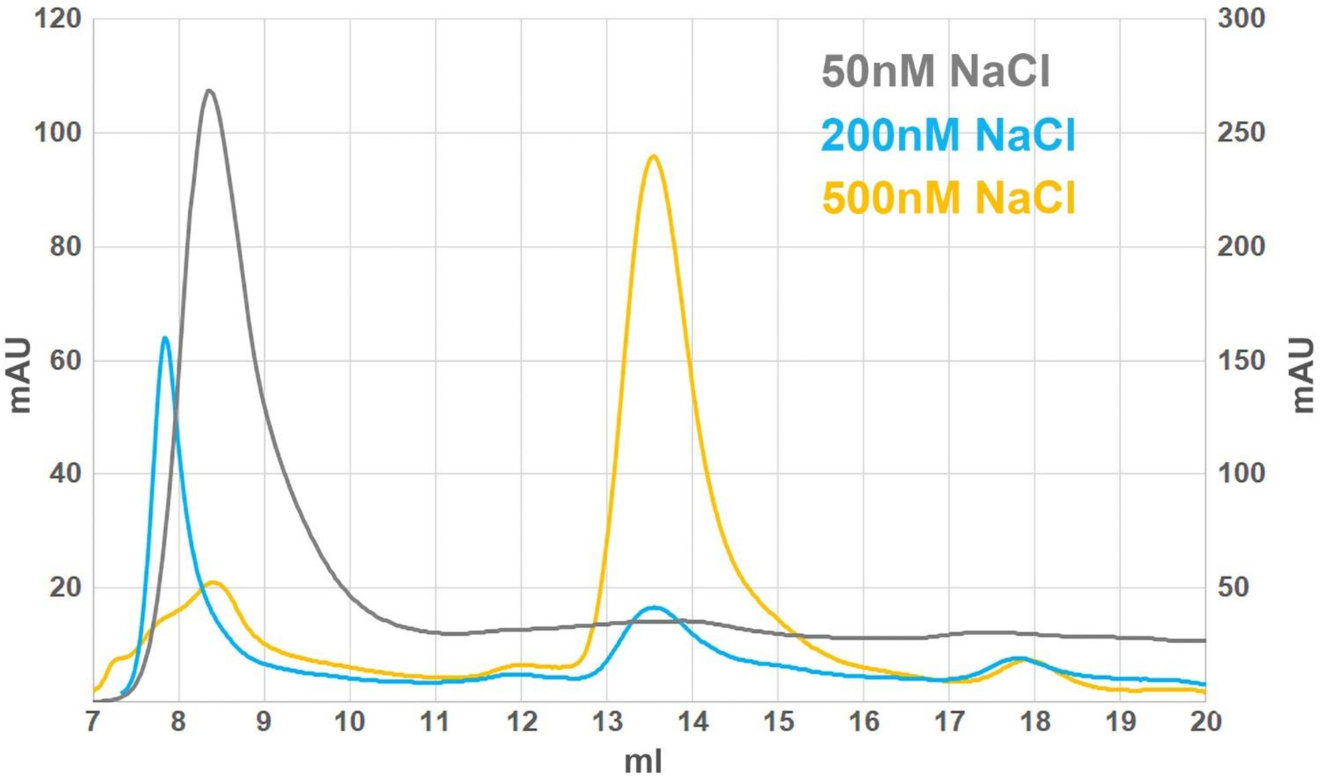

Supplementary Figure 2. SEC analysis of $GP46_{F13}$ protein fractions eluted from the Superdex 200 Increase 10/300 column at 500 mM **(yellow)**, 200 mM **(blue)** and 50 mM **(gray)** NaCl concentration.
